# A single nucleotide polymorphism in the interferon-γ gene (*IFNG* +874 T/A) is associated with susceptibility to tuberculosis

**DOI:** 10.18632/oncotarget.17304

**Published:** 2017-04-20

**Authors:** Zhang Wei, Shen Wenhao, Mi Yuanyuan, Li Yang, Zhou Daming, Xian Jiangchun, Jiang Jijun

**Affiliations:** ^1^ Department of Infectious Disease, Taizhou People’s Hospital, Taizhou, China; ^2^ Department of Oncology, Taizhou People’s Hospital, Taizhou, China; ^3^ Department of Urology, Third Affiliated Hospital of Nantong University, Wuxi, China

**Keywords:** Interferon-γ, pulmonary tuberculosis, extra-pulmonary tuberculosis, polymorphism, meta-analysis, Immunology and Microbiology Section, Immune response, Immunity

## Abstract

Interferon-γ (Interferon gamma, IFNG) is an important cytokine involved in providing resistance to mycobacterial diseases. Common variants of *IFNG*, such as *IFNG* +874 T/A(rs2430561), may be related to tuberculosis susceptibility, but this association has not been consistently observed. We performed an updated meta-analysis to evaluate the association between the *IFNG +874 T/A* (rs2430561) polymorphism and tuberculosis susceptibility. PubMed and SinoMed databases were searched up to October 2016, and odds ratios (OR) and 95% confidence intervals (CI) were used to assess the association strength. Based on search criteria for manuscripts reporting tuberculosis susceptibility and its relationship with the *IFNG* +874 T/A(rs2430561)polymorphism, 42 case-control studies from 39 different articles were retrieved. Significantly positive, decreased, and protective associations were found between the *IFNG* +874 T/A(rs2430561)polymorphism and tuberculosis risk in five genetic models. Moreover, in the stratified subgroup analysis, a protective relationship was detected in four different ethnicities and sources of the control groups. Furthermore, the *IFNG* +874 T/A(rs2430561)polymorphism played an important role in protecting individuals from both pulmonary tuberculosis and extra-pulmonary tuberculosis. Our meta-analysis suggests that the *IFNG* +874 T/A(rs2430561)polymorphism is potentially associated with tuberculosis susceptibility and may be used as a predictive biomarker. Further studies with larger sample sizes and consideration of gene-environment interactions should be conducted to elucidate the role of *IFNG* +874 T/A(rs2430561) polymorphism in tuberculosis susceptibility.

## INTRODUCTION

Tuberculosis (TB) is a chronic infectious disease caused by *Mycobacterium tuberculosis* (MTB). Data from the global tuberculosis report estimated a worldwide incidence of 8.7 million cases, most of which occurred in Asia and Africa [1]. One-third of the world's population is potentially infected with *M. tuberculosis*, but only 10% of the infected individuals eventually develop clinical TB, indicating that the host's genetic and other factors (such as alcohol consumption, malnutrition, and human immunodeficiency virus infection) may play essential and complex roles in determining susceptibility and progression to tuberculosis [2–4]. An increasing number of studies have focused on the relationship between genetic variations of several genes and TB susceptibility [5, 6].

Currently, the gene encoding interferon-γ (*IFNG*) is the most extensively studied gene regarding tuberculosis susceptibility. Human *IFNG* is located on chromosome 12 (12q14) and has four exons spanning approximately 6 kb. Interferon-γ (IFN-γ), which is encoded by *IFNG* and produced by T helper 1 (Th1) lymphocytes, is upregulated and secreted as a major cytokine to activate macrophages, and is critical for the control of *M. tuberculosis* infection [7]. Cases of active TB are characterized by decreased production of IFN-γ from the peripheral blood mononuclear cells compared with that observed in latent infection. In addition, local and systemic IFN-γ levels correlate with the severity of the disease [8].

Lio et al. first noted a significant association between the +874A/T polymorphism (rs2430561) in *IFNG* and protection against TB in Sicilia (*P* < 0.05) [9]. The SNP +874 (A/T) is located at the 5′-end of a CA repeat in the first intron of the human *IFNG* [10]. The +874 T-allele is linked to the 12 CA repeats, whereas the A-allele is linked to the non-twelve CA repeats [11]. The specific sequence of the T-allele provides a binding site for the transcription factor nuclear factor-κB [12, 13]. As nuclear factor-κB induces IFN-γ expression, the presence of the T-allele correlates with high IFN-γ expression, whereas that of the A-allele correlates with low expression.

Many epidemiologic studies, including meta-analyses [14, 15], suggested associations between *IFNG* *+874 T/A* (rs2430561) polymorphism and the risk of developing TB. However, the data were inconsistent, partly because of differences in study populations and case ascertainment criteria, or small sample sizes with a high rate of false-positives and limited power of detecting modest associations. Considering the important role of *IFNG* *+874 T/A* (rs2430561) polymorphism in TB development, we studied all eligible case-control studies that included characteristics such as ethnicity of the patients, types of tuberculosis, and control sources.

## RESULTS

### Study characteristics

**Figure 1:**
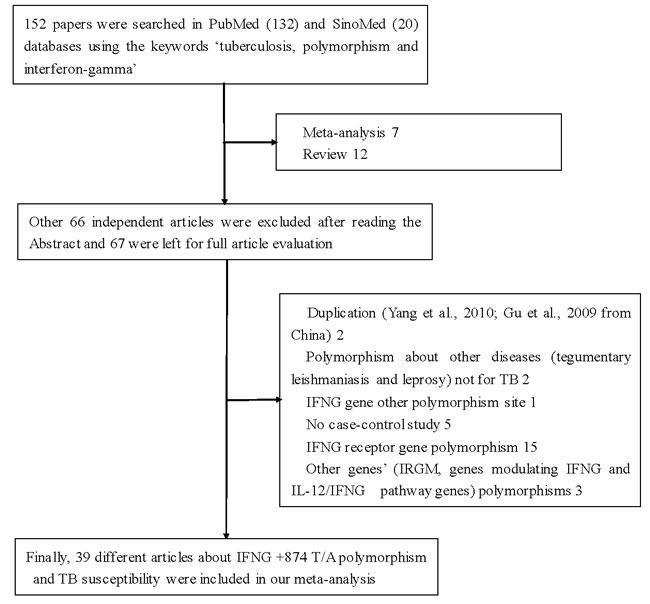
Flowchart illustrating the search strategy used to identify association studies for *IFNG +874 T/A* (rs2430561) polymorphism and tuberculosis risk

**Figure 2:**
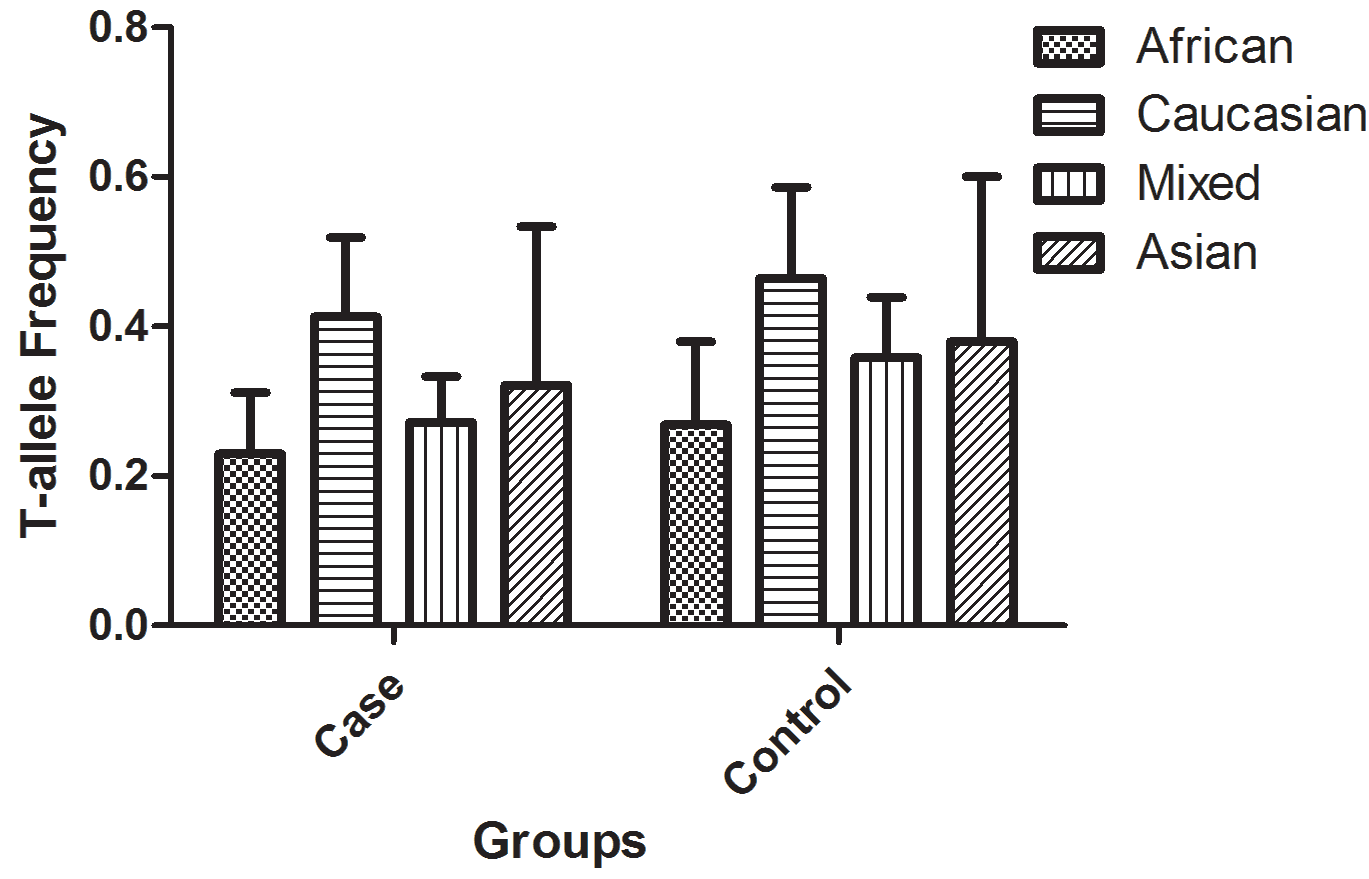
T-allele frequencies for the *IFNG +874 T/A* (rs2430561) polymorphism among cases/controls stratified by ethnicity Vertical line, T-allele frequency; Horizontal line, ethnicity type.

Using various combinations of key terms, 152 article titles were retrieved by a literature search using the PubMed and SinoMed databases. As shown in Figure 1, 66 articles were excluded after screening the abstracts of the manuscripts. The full texts were evaluated, and 47 additional articles were excluded because of the following reasons: duplication (two), meta-analysis (seven), review (twelve), discussion on polymorphism in other diseases (tegumentary leishmaniasis and leprosy) (two), other SNPs in *IFNG* (one), IFNG receptor gene polymorphisms (fifteen), and other genes (three), and lacked case-control study (five). Finally, 39 different articles [9, 16–53] concerning *IFNG* *+874 T/A* (rs2430561) polymorphism and TB susceptibility were included in our meta-analysis (Table 1). Overall, 42 case-control studies with 8,574 cases and 9,011 controls were retrieved based on the search criteria. The controls were mainly healthy populations. First, we checked the Minor Allele Frequency (MAF) reported for the five main worldwide populations in the 1000 Genomes Browser: East Asian, 0.8413; European, 0.5378; African, 0.8327; American, 0.7464; and South Asian, 0.6104. The MAF in our analysis was 0.324 and 0.382 in the case and control group, respectively, both lower than the results in the 1000 Genomes Browser database. Then, the frequency of the T-allele was found to be significantly higher in case individuals of Caucasian ethnicity than in those of African or mixed ethnicities (41.3% vs. 22.9%, *P* = 0.005, or 41.3% vs. 27.2%, *P* = 0.017, respectively). A similar trend was found in the control group (Figure 2). Except for seven studies [16, 21, 26, 30, 36, 38, 43], the distribution of genotypes in all the controls was in agreement with Hardy-Weinberg equilibrium (HWE).

**Table 1:** Basic information for included studies of the association between *IFNG +874 T/A (rs2430561*) polymorphism site and tuberculosis susceptibility

First author	Year	Origin	Source of Control	Ethnicity	Type	Sample size	Case	Control	Case					Control		Control						Methods		NOS
Ref No									TT	TA	AA	MAF	HWE	TT	TA	AA		MAF		HWE				
Amim*[34]	2008	Brazil	HB	Mixed	TB	<100	93	266	15	25	53	0.296	0.0006	67	88	111	0.417		0.001		PCR-ARMS		6	
Anand[21]	2010	India	HB	Asian	PTB	<100	62	66	7	27	28	0.331	0.898	8	25	33	0.311		0.35		PCR-ASP		6	
Ansari*[53]	2009	Pakistan	HB	Asian	PTB	>100	111	188	27	47	37	0.455	0.123	25	87	76	0.364		0.98		PCR-ARMS		7	
Ansari*[53]	2009	Pakistan	HB	Asian	EPTB	<100	77	188	12	36	29	0.389	0.881	25	87	76	0.364		0.98		PCR-ARMS		7	
Anuradha*[27]	2008	India	PB	Asian	Children TB	<100	25	90	5	13	7	0.46	0.815	29	42	19	0.555		0.6		PCR		6	
Asgharzadeh[64]	2016	Iran	PB	Asian	TB	>100	124	200	10	42	72	0.25	0.281	28	81	91	0.342		0.15		PCR-ARMS		8	
Bhanothu[47]	2015	India	HB	Asian	EPTB	>100	175	100	45	87	43	0.506	0.941	29	62	9	0.6		0.003		ARMS-MG/ MP-PCR		6	
Cooke*[36]	2006	UK	PB	African	PTB	>100	667	597	20	159	488	0.149	0.115	13	166	415	0.162		0.45		PCR-ARMS		6	
Ding*[26]	2008	China	PB	Asian	TB	>100	301	310	19	89	193	0.211	0.052	32	114	164	0.287		0.07		sequencing		8	
Etokebe*[40]	2005	Croatia	HB	Caucasian	TB	>100	242	519	53	122	67	0.471	0.637	103	282	134	0.47		0.04		PCR-ASP		6	
Fitness*[48]	2004	Malawi	PB	African	TB	>100	451	703	12	134	305	0.175	0.549	17	219	467	0.179		0.14		PCR-ARMS		8	
Gutlapalli[32]	2016	India	HB	Asian	TB	>100	247	129	73	123	51	0.544	0.951	60	58	11	0.689		0.56		PCR-ARMS		6	
Hashemi[20]	2011	Iran	PB	Asian	PTB	>100	142	166	18	84	40	0.422	0.011	33	111	22	0.533		0.001		PCR-ARMS		7	
Hu[45]	2015	China	PB	Asian	TB	>100	360	480	36	124	200	0.272	0.013	67	201	212	0.349		0.08		PCR-ARMS		7	
Hwang*[50]	2007	Korea	PB	Asian	PTB	<100	80	80	1	13	66	0.094	0.696	0	21	59	0.131		0.17		PCR-ARMS		7	
Leandro[43]	2013	Brazil	PB	Mixed	TB	>100	172	179	22	78	72	0.354	0.902	26	91	62	0.399		0.42		PCR-ARMS		8	
Lee[44]	2015	Taiwan	PB	Asian	TB	>100	200	202	131	56	13	0.795	0.046	144	54	4	0.846		0.68		TaqMan		8	
Lio[9]	2002	Italy	PB	Caucasian	PTB	<100	45	97	4	30	11	0.422	0.013	25	47	25	0.5		0.06		PCR-ARMS		8	
Lopez- Maderuelo*[39]	2003	Spain	PB	Caucasian	PTB	>100	113	100	11	40	62	0.274	0.238	19	50	31	0.44		0.88		PCR		6	
Ma*[49]	2007	China	PB	Asian	PTB	<100	60	60	2	10	48	0.117	0.138	8	20	32	0.3		0.11		PCR-SSP		6	
Mabunda[29]	2015	Brazil	HB	Mixed	PTB	>100	101	360	3	32	66	0.188	0.708	21	136	203	0.247		0.77		TaqMan		7	
Moran*[30]	2007	USA	PB	African	TB	>100	240	174	9	87	144	0.219	0.348	11	65	98	0.25		0.96		sequencing		7	
Moran*[30]	2007	USA	PB	Caucasian	TB	<100	161	64	24	92	45	0.435	0.039	16	31	17	0.492		0.8		sequencing		7	
Moran*[30]	2007	USA	PB	Caucasian	TB	<100	319	98	24	96	199	0.226	0.013	10	26	62	0.235		0.01		sequencing		7	
Mosaad[42]	2010	Egypt	HB	Caucasian	TB	>100	110	118	32	60	18	0.563	0.253	52	60	6	0.695		0.03		PCR-ARMS		6	
Onay*[22]	2010	Turkey	HB	Caucasian	Children TB	<100	40	67	9	22	9	0.5	0.527	16	35	16	0.5		0.71		PCR-ARMS		6	
Oral*[35]	2006	Turkey	PB	Caucasian	TB	<100	81	50	21	29	31	0.438	0.014	8	21	21	0.37		0.48		PCR-SSP		8	
Rossouw*[38]	2003	UK	PB	African	TB	>100	313	235	25	102	186	0.243	0.078	26	98	111	0.319		0.53		PCR		6	
Sallakci*[33]	2007	Turkey	HB	Caucasian	TB	>100	361	115	50	182	129	0.391	0.262	26	58	31	0.478		0.91		sequencing		6	
Selma*[25]	2011	Tunisia	PB	African	TB	>100	223	150	38	85	100	0.361	0.009	22	86	42	0.433		0.04		PCR-RFLP		7	
Selvaraj*[52]	2008	India	HB	Asian	PTB	>100	160	178	20	72	68	0.35	0.889	23	76	79	0.342		0.48		PCR-RFLP		7	
Shen[28]	2013	China	PB	Asian	Children TB	>100	189	164	136	53	0	0.859	0.024	133	29	2	0.899		0.77		sequencing		6	
Tso*[37]	2005	Hong Kong	HB	Asian	TB	>100	385	451	17	101	267	0.175	0.068	55	190	206	0.332		0.28		sequencing		6	
Vallinoto*[23]	2010	Brazil	PB	Mixed	TB	>100	162	156	11	58	93	0.247	0.635	24	86	46	0.429		0.12		PCR-ASO		7	
Vidyarani*[31]	2006	India	HB	Asian	PTB	>100	129	127	14	54	61	0.318	0.694	20	52	55	0.362		0.2		PCR-ARMS		6	
Wang*[54]	2011	China	HB	Asian	PTB	>100	273	297	3	78	192	0.154	0.107	3	84	210	0.151		0.08		PCR-RFLP		6	
Wang*[24]	2010	China	PB	Asian	PTB	>100	521	526	14	80	427	0.104	<0.001	10	91	425	0.105		0.06		PCR-RFLP		6	
Wit*[51]	2011	South Africa	PB	Mixed	TB	>100	500	315	40	194	266	0.274	0.579	33	124	158	0.301		0.24		PCR-RFLP		6	
Wu*[41]	2008	China	PB	Asian	PTB	<100	61	122	1	10	50	0.098	0.554	2	24	96	0.114		0.72		PCR-RFLP		8	
Yang*[57]	2010	China	HB	Asian	PTB	>100	189	191	6	41	142	0.14	0.168	3	49	139	0.143		0.57		PCR-SSP		6	
Zhong[55]	2014	China	HB	Asian	PTB	>100	142	166	4	26	112	0.119	0.117	7	49	110	0.189		0.61		TaqMan		6	
Zhuang[56]	2009	China	HB	Asian	PTB	>100	167	167	12	53	102	0.231	0.172	37	70	60	0.431		0.06		PCR-SSP		5	

HWE: Hardy–Weinberg equilibrium; HB: hospital-based; PB: population-based; TB: tuberculosis; PTB: pulmonary TB, EPTB: extra-pulmonary TB; PCR-FLIP: polymerase chain reaction and restrictive fragment length polymorphism; PCR-ARMS: polymerase chain reaction-amplification refractory mutation specific; PCR-ASP: allele specific primers by polymerase chain reaction, PCR-SSP: polymerase chain reaction-sequence specific primer; PCR-ASO: polymerase chain reaction-allele-specific oligonucleotide; ARMS-MG/MP-PCR: Amplification refractory mutation system-multi gene/multi primer polymerase chain reaction; NOS: Newcastle-Ottawa Scale; *: studies reported in previous meta-analysis; bold: these nine studies including both PTB and EPTB.

### Quantitative synthesis

**Figure 3:**
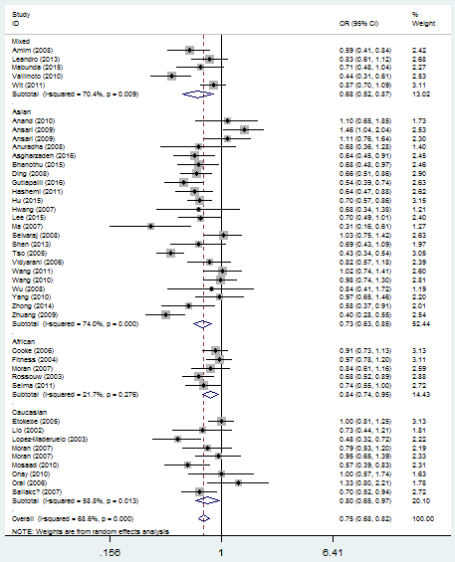
Forest plot of tuberculosis risk associated with *IFNG +874 T/A* (rs2430561) polymorphism (additive model, TA vs. AA) in the whole The squares and horizontal lines correspond to the study-specific OR and 95% CI. The area of the squares reflects the weight (inverse of the variance). The diamond represents the summary OR and 95% CI.

In the overall analysis, a significant protective association could be observed between TB risk and the variant genotype of *IFNG* *+874 T/A* (rs2430561) polymorphism in different genetic models. For example, for the additive model (TA vs. AA), odds ratios (OR) = 0.71, 95% confidence intervals (CI) = 0.63-0.80, *P-*value of heterogeneity test (*P*_h_) = 1×10^-6^, and in the dominant model, OR = 0.68, 95% CI = 0.60-0.77, *P*_h_ = 1×10^-6^ (Figure 3, Table 2). If some studies that do not conform to HWE were removed, these associations were not affected.

**Table 2:** Total and stratified subgroup analysis for *IFNG +874 T/A(rs2430561*) polymorphism site and tuberculosis susceptibility

Variables	N	Case/	Allelic effect		Additive model (TA vs. AA)/(TT vs. AA)		Dominant model		Recessive model		Overdominant model
		Control	OR(95%CI) *Ph P*		OR(95%CI) *Ph P*		OR(95%CI) *Ph P*		OR(95%CI) *Ph P*		OR(95%CI) *Ph P*
Total	42	8574/9011	0.75 (0.68-0.82) 1×10^-6^ 1×10^-6^		0.71 (0.63-0.80) 1×10^-6^ 1×10^-6^/0.60 (0.49-0.73) 1×10^-6^ 1×10^-6^		0.68 (0.60-0.77) 1×10^-6^ 1×10^-6^		0.72 (0.62-0.83) 0.002 1×10^-6^		0.83 (0.76-0.92) 1×10^-6^ 1×10^-6^
HWE	35	7270/7594	0.75 (0.67-0.84) 1×10^-6^ 1×10^-6^		0.74 (0.65-0.83) 1×10^-6^ 1×10^-6^/0.62 (0.49-0.78) 1×10^-6^ 1×10^-6^		0.71 (0.62-0.81) 1×10^-6^ 1×10^-6^		0.70 (0.59-0.83) 0.004 1×10^-6^		0.85 (0.76-0.94) 0.001 0.002
Ethnicity											
Asian	23	4180/4648	0.73 (0.63-0.84) 1×10^-6^ 1×10^-6^		0.68 (0.57-0.81) 1×10^-6^ 1×10^-6^/0.58 (0.42-0.81) 1×10^-6^ 0.001		0.65 (0.54-0.79) 1×10^-6^1×10^-6^		0.70 (0.57-0.87) 0.004 0.001		0.83 (0.72-0.95) 0.005 0.006
Caucasian	9	142/1228	0.79 (0.65-0.97) 0.013 0.025		0.83 (0.63-1.09) 0.095 0.181/0.60 (0.39-0.92) 0.014 0.019		0.77 (0.58-1.03) 0.036 0.078		0.70 (0.49-0.96) 0.003 0.028		0.99 (0.84-1.17) 0.135 0.926
African	5	1894/1859	0.84 (0.75-0.95) 0.276 0.004		0.74 (0.57-0.94) 0.026 0.016/0.79 (0.58-1.09) 0.276 0.152		0.75 (0.60-0.94) 0.048 0.013		0.97 (0.72-1.30) 0.392 0.833		0.76 (0.61-0.95) 0.047 0.017
Mixed	5	1028/1276	0.67 (0.52-0.84) 0.009 0.003		0.64 (0.45-0.92) 0.012 0.015/0.53 (0.42-0.72) 0.009 1×10^-6^		0.61 (0.42-0.87) 0.004 0.007		0.64 (0.48-0.85) 0.545 0.002		0.75 (0.57-0.97) 0.094 0.030
Source of Control											
HB	18	3064/3693	0.76 (0.64-0.91) 1×10^-6^ 0.003		0.72 (0.59-0.87) 1×10^-6^ 0.001/0.59 (0.41-0.86) 1×10^-6^ 0.006		0.69 (0.54-0.86) 1×10^-6^ 0.001		0.72 (0.55-0.94) 1×10^-6^ 0.016		0.85 (0.73-0.98) 0.030 0.023
PB	24	5510/5318	0.74 (0.68-0.82) 0.003 1×10^-6^		0.70 (0.61-0.81) 0.001 1×10^-6^/0.60 (0.49-0.75) 0.030 1×10^-6^		0.68 (0.59-0.79) 1×10^-6^ 1×10^-6^		0.71 (0.63-0.82) 0.246 1×10^-6^		0.82 (0.72-0.94) 0.001 0.004
TB type											
PTB	25	4244/5107	0.73 (0.64-0.83) 1×10^-6^ 1×10^-6^		0.68 (0.58-0.80) 0.001 1×10^-6^/0.60 (0.44-0.81) 1×10^-6^ 0.001		0.66 (0.55-0.78) 1×10^-6^ 1×10^-6^		0.71 (0.57-0.90) 0.006 0.004		0.80 (0.70-0.91) 0.008 0.001
EPTB	10	984/1910	0.66 (0.55-0.79) 0.040 1×10^-6^		0.55 (0.39-0.77) 0.027 1×10^-6^/0.45 (0.27-0.75) 0.007 0.002		0.51 (0.36-0.73) 0.008 1×10^-6^		0.66 (0.49-0.89) 0.075 0.006		0.88 (0.66-1.17) 0.007 0.347
Sample size											
<100	12	1104/1248	0.82 (0.67-0.99) 0.043 0.047		0.89 (0.72-1.09) 0.323 0.262/0.70 (0.53-0.94) 0.283 0.018		0.83 (0.69-1.01) 0.161 0.056		0.71 (0.54-0.92) 0.253 0.011		0.82 (0.67-0.99) 0.043 0.047
>100	30	7470/7763	0.73 (0.66-0.81) 1×10^-6^ 1×10^-6^		0.67 (0.59-0.76) 0.000 0.000/0.56 (0.45-0.72) 0.000 0.000		0.64 (0.56-0.74) 1×10^-6^ 1×10^-6^		0.71 (0.61-0.84) 0.001 1×10^-6^		0.81 (0.73-0.89) 0.001 1×10^-6^
Genotype methods											
PCR-SSP	4	497/468	0.64 (0.33-1.22) 1×10^-6^ 0.177		0.60 (0.45-0.80) 0.109 0.001/0.58 (0.14-2.30) 1×10^-6^ 0.437		0.57 (0.31-1.08) 0.002 0.088		0.69 (0.21-2.33) 0.002 0.558		0.68 (0.51-0.90) 0.555 0.007
PCR	3	451/425	0.62 (0.50-0.77) 0.346 1×10^-6^		0.57 (0.42-0.77) 0.356 1×10^-6^/0.46 (0.29-0.72) 0.439 0.001		0.54 (0.41-0.72) 0.291 1×10^-6^		0.59 (0.38-0.90) 0.689 0.015		0.68 (0.51-0.90) 0.390 0.007
sequencing	7	1956/1376	0.69 (0.55-0.86) 0.003 0.001		0.78 (0.55-1.11) 0.001 0.173/0.45 (0.34-0.59) 0.153 1×10^-6^		0.71 (0.50-1.00) 0.001 0.050		0.52 (0.41-0.66) 0.729 1×10^-6^		0.97 (0.69-1.37) 1×10^-6^ 0.858
PCR-ARMS	15	2845/3545	0.78 (0.68-0.90) 0.001 0.001		0.77 (0.69-0.86) 0.105 1×10^-6^/0.65 (0.46-0.92) 1×10^-6^ 0.014		0.73 (0.61-0.87) 0.005 0.001		0.76 (0.59-0.98) 0.012 0.034		0.87 (0.79-0.97) 0.350 0.014
PCR-RFLP	6	1738/1588	0.91 (0.80-1.03) 0.640 0.131		0.84 (0.65-1.08) 0.042 0.177/0.87 (0.64-1.18) 0.793 0.360		0.87 (0.75-1.01) 0.114 0.072		0.98 (0.73-1.31) 0.771 0.902		0.85 (0.67-1.09) 0.045 0.204
TaqMan	3	443/728	0.67 (0.53-0.84) 0.771 0.001		0.59 (0.42-0.83) 0.377 0.002/0.39 (0.19-0.79) 0.714 0.009		0.57 (0.41-0.79) 0.357 0.001		0.72 (0.49-1.05) 0.795 0.084		0.79 (0.60-1.04) 0.149 0.095
PCR-ASP	2	284/585	1.02 (0.83-1.24) 0.761 0.871		0.93 (0.67-1.29) 0.359 0.675/1.03 (0.68-1.55) 0.997 0.891		0.96 (0.71-1.31) 0.464 0.809		1.11 (0.78-1.58) 0.725 0.570		0.91 (0.69-1.20) 0.318 0.505

*P*_h_: value of *Q*-test for heterogeneity test; *P*: *Z*-test for the statistical significance of the OR. HWE: Except for seven studies[20,25,30,34,40,42,47], the distribution of genotypes in the controls in remaining 35 studies was in agreement with HWE, and it reports the meta-analysis results of studies showing no deviation from the HWE.

**Figure 4:**
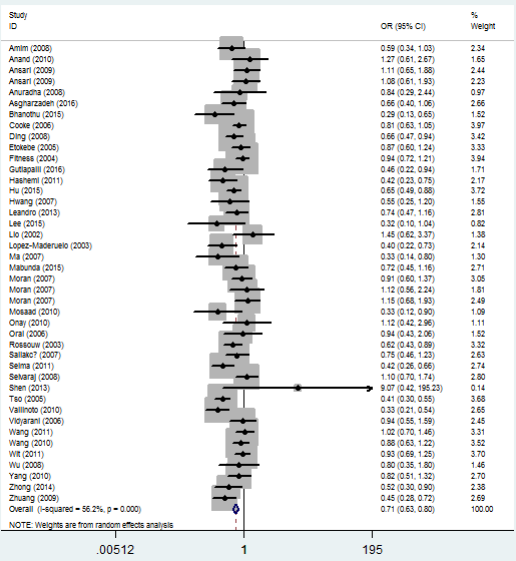
Forest plot of tuberculosis risk associated with *IFNG +874 T/A* (rs2430561) polymorphism (the allelic effect) by ethnicity The squares and horizontal lines correspond to the study-specific OR and 95% CI. The area of the squares reflects the weight (inverse of the variance). The diamond represents the summary OR and 95% CI.

**Figure 5:**
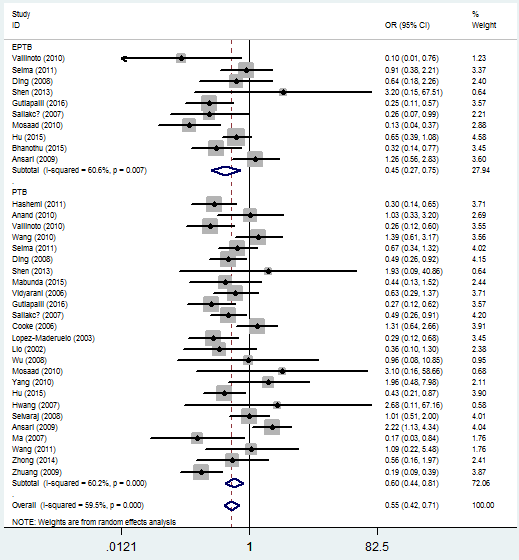
Forest plot of tuberculosis risk associated with *IFNG +874 T/A* (rs2430561) polymorphism (dominant model) by source of control The squares and horizontal lines correspond to the study-specific OR and 95% CI. The area of the squares reflects the weight (inverse of the variance). The diamond represents the summary OR and 95% CI.

In the ethnicity type subgroup, this pattern of positive association was maintained in the whole genetic models. For example, for the allelic effect, OR = 0.84, 95% CI = 0.75-0.95, *P*_h_ = 0.276 for Africans; OR = 0.73, 95% CI = 0.63-0.84, *P*_h_ = 1×10^-6^ for Asians; OR = 0.79, 95% CI = 0.65-0.97, *P*_h_ = 0.013 for Caucasians; OR = 0.67, 95% CI = 0.52-0.84, *P*_h_ = 0.009 for mixed ethnicity (Figure 4, Table 2). Similar results were detected in the source of control analysis. For example, in the dominant model, OR = 0.69, 95% CI = 0.54-0.86, *P*_h_ = 1×10^-6^ for HB, and OR = 0.68, 95% CI = 0.59-0.79, *P*_h_ = 1×10^-6^ for PB (Figure 5, Table 2).

**Figure 6:**
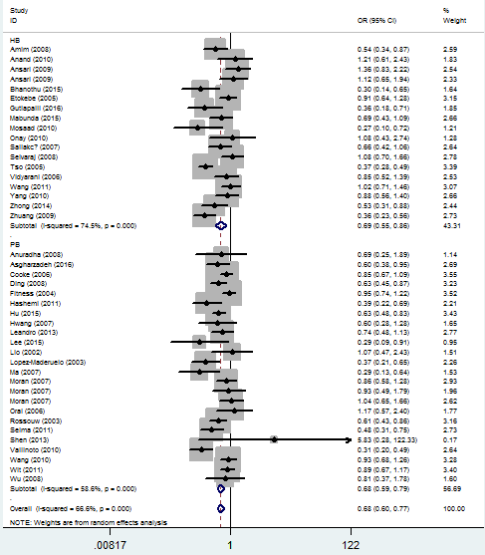
Forest plot of tuberculosis risk associated with *IFNG +874 T/A* (rs2430561) polymorphism (additive model, TT vs. AA) by type of tuberculosis The squares and horizontal lines correspond to the study-specific OR and 95% CI. The area of the squares reflects the weight (inverse of the variance). The diamond represents the summary OR and 95% CI.

In the stratified analysis by TB type, significant associations were found with both pulmonary tuberculosis (PTB) and extra-pulmonary tuberculosis (EPTB) risks and *IFNG* *+874 T/A* (rs2430561) polymorphism in the five genetic models. For example, for the additive model (TT vs. AA), OR = 0.60, 95% CI = 0.44-0.81, *P*_h_ = 1×10^-6^ for PTB, and OR = 0.45, 95% CI = 0.27-0.75, *P*_h_ = 0.007 for EPTB (Figure 6, Table 2).

To improve our study, finally we made stratified subgroup analyses according to genotyping methods and sample size. Some positive associations were also found in this section (Table 2).

### Bias diagnosis and sensitivity analysis

**Figure 7:**
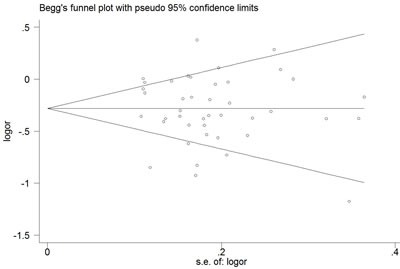
Begg's funnel plot for publication bias test (additive model, TA vs. AA) Each point represents a separate study for the indicated association. Log [OR], natural logarithm of OR. Horizontal line, mean effect size.

**Figure 8:**
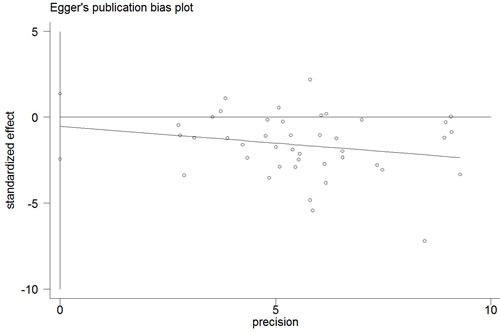
Egger's publication bias plot (additive model, TA vs. AA)

**Figure 9:**
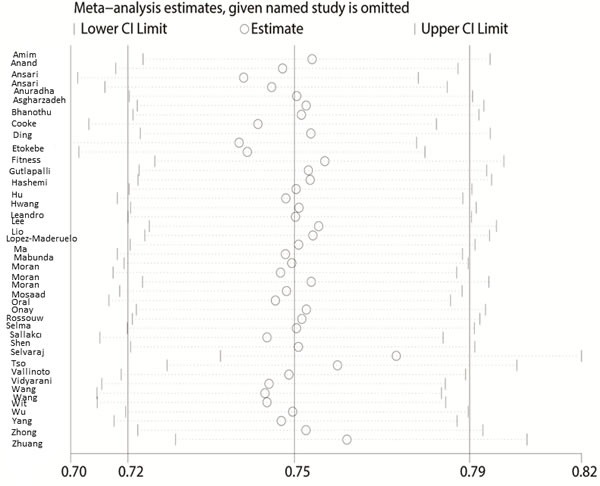
Sensitivity analysis between IFNG +874 T/A (rs2430561) polymorphism and tuberculosis risk (the allelic effect)

The Begg's funnel plot and Egger's test were performed to access the publication bias of the literature. The shape of the funnel plot did not reveal any obvious asymmetry, and the Egger's test suggested the absence of publication bias. For example, in the additive model (TA vs. AA) analysis, z = 1.11, *P* = 0.269 for Begg's test and t = −0.87, *P* = 0.389 for Egger's test (Figures 7, 8, Table 3). We used a sensitivity analysis to determine whether modification of the meta-analysis inclusion criteria affected the results. No single study qualitatively influenced the summary ORs, as indicated by the sensitivity analysis (for example, the allelic effect, Figure 9).

**Table 3:** Publication bias tests (Begg's funnel plot and Egger's test for publication bias test) for *IFNG +874 T/A(rs2430561*) polymorphism

Egger's test						Begg's test	
Genetic type	Coefficient	Standard error	*t*	*P* value	95%CI of intercept	*z*	*P* value
Allelic effect	-0.521	0.946	-0.55	0.585	(-2.432,1.390)	0.26	0.795
Additive model (TA *vs*. AA)	-0.443	0.509	-0.87	0.389	(-1.473, 0.587)	1.11	0.269
Additive model (TT *vs*. AA)	-0.08	0.345	-0.23	0.817	(-0.778, 0.617)	0.09	0.931
Dominant model	-0.414	0.545	-0.76	0.452	(-1.516, 0.688)	1	0.319
Recessive model	-0.108	0.381	0.28	0.779	(-0.663, 0.878)	0.76	0.448
Overdominant model	-0.559	0.687	-0.81	0.42	(-1.945, 0.829)	0.89	0.347

## DISCUSSION

To understand the pathogenesis of TB and better predict individual risk, it is important to identify the SNPs that affect the function of genes contributing to TB susceptibility. Genome-wide association studies (GWAS) allow genotyping of frequent genetic polymorphisms in the genome without assuming the genomic location of the causal variants. As most of the genome has been surveyed, GWAS eliminates the disadvantages associated with the single polymorphism or candidate gene approach where only a few polymorphisms are investigated [54]. However, GWAS may identify false associations, and therefore, replication or validation of the results in independent populations is essential [55]. A Wellcome Trust Case-Control Consortium-based study initiated GWAS that considered up to 2,000 cases and controls each for tuberculosis and malaria. This study identified *NRAMP1*, *IFNG*, *NOS2A*, *MBL*, *VDR*, and certain TLR-associated genes as important for susceptibility to TB and malaria, and the list has been growing ever since [55–57].

Lio et al. [9] were the first to investigate the association between *IFNG* *+874 T/A* (rs2430561) polymorphism and the risk of developing TB. Since then, other researchers have reproduced their work on *IFNG* in different populations and for two disparate types of TB. However, the results were confounding, even within populations. Meta-analysis provides a means for effectively increasing the size of the sample by pooling data from individual correlation studies, thus enhancing the statistical power of the analysis to estimate genetic effects [58]. We used this method to demonstrate statistically significant genetic associations.

To the best of our knowledge, this is the first systematic meta-analysis exploring the associations between *IFNG* polymorphism and TB risk. Our analysis involved about 8,574 individuals with TB and 9,011 controls. The main discovery of our study was that the T-allele of the *IFNG* *+874 T/A* (rs2430561) polymorphism showed a protective effect for TB susceptibility, which was consistent with the observation that the presence of the T-allele correlates with high IFN-γ expression and increased resistance to *M. tuberculosis* infection, whereas the A-allele correlates with low expression. The highlight of our article is that we classified TB into PTB and EPTB, which assisted in the analysis of effective target sites for different types of TB, and thereby, we provided a novel concept compared to the previously published meta-analyses [14, 15].

Although considerable efforts and resources have been invested in examining the possible associations between IFN gene polymorphisms and TB risk, certain inherent limitations exist. First, although we collected all eligible studies, the sample sizes of these studies were not large enough to provide adequate statistical power to evaluate the association between this polymorphism and TB risk, particularly for certain ethnicities. Thus, not only was there an increased likelihood of type I/II error, but also a lack of sufficient statistical power to evaluate the association between these polymorphisms and tuberculosis risk. Second, because of the complex nature of the immune system and the polygenic nature of complex diseases, such as TB, it has become increasingly evident that gene-gene interactions play a far more important part in an individual's susceptibility to tuberculosis than single polymorphisms would on their own [59]. Specific environmental and lifestyle factors, including age, sex, smoking habits, familial history, and disease stage, may possibly alter the associations between *IFNG* polymorphisms and TB. Therefore, it is necessary to evaluate the roles of specific environmental factors and lifestyles in disease prognosis.

In spite of these drawbacks, our meta-analysis provided several advantages. First, a substantial number of cases and controls were pooled from different studies, which significantly increased the statistical power of the analysis. Second, no publication bias was found and the Newcastle-Ottawa Scale (NOS) scores of the included studies indicated high quality. Third, our study contained the highest number of individuals so far, which conferred increased statistical power and credibility. The power of this meta-analysis of *+874 T/A* (rs2430561) polymorphism was greater than 0.95, which suggested that the data was trustworthy.

In conclusion, our present meta-analysis suggests that the *+874 T/A* (rs2430561) polymorphism in *IFNG* may be associated with the risk of developing TB. Further studies that consider additional gene-environment interactions and are based on larger sample sizes should be conducted to elucidate the role of *IFNG* polymorphisms in the etiology and clinical characteristics of TB development.

## MATERIALS AND METHODS

### Search strategy

We searched the PubMed and SinoMed (http://www.sinomed.ac.cn/) databases through October 2016, using the keywords “tuberculosis,” “TB,” “polymorphism,” and “IFN-gamma.” One hundred and fifty-two articles were retrieved, 39 of which satisfied our inclusion criteria. We also screened references of the retrieved articles and reviewed all articles via a manual search.

### Inclusion and exclusion criteria

For inclusion, studies were required to (i) assess the association between TB risk and *IFNG* *+874 T/A* (rs2430561) polymorphism; (ii) be case-controlled, and (iii) contain all three kinds of genotypes (TT, TA, and AA) for cases and controls. Studies were excluded if they (i) included no control population; (ii) did not report genotype frequency data, and (iii) were duplicated publications.

### Quality score assessment

The NOS [60] was selected to assess the quality of each study. This measure assesses aspects of the methodologies used in observational studies, which are related to the study quality, including selection of cases, comparability of populations, and ascertainment of exposure to risks. The NOS rating ranges from zero stars (worst) to nine stars (best). Studies with a score of seven stars or greater were considered to be of high quality.

### Data extraction

Two authors independently extracted the data that complied with the selection criteria. These data included the first author's last name, year of publication, country of origin, ethnicity of the study population, TB type, genotypes of both the case and control groups, source of control, HWE/ MAF in both case and control groups, and genotyping method. Ethnicity was categorized as Caucasian, Asian, African, and mixed population. The control subgroups were population-based (PB) and hospital-based (HB). The TB type included PTB and EPTB. All included studies were classified as larger (both number of case and control ≥ 100 samples) and medium-sized studies. The NOS score was also shown in this section.

### Statistical analysis

Based on the genotype frequencies for cases and controls, OR with 95% CI were used to measure the strength of the associations between the *+874 T/A* (rs2430561) single nucleotide polymorphism (SNP) in *IFNG* and TB risk. The statistical significance of the OR was determined with the *Z*-test. The heterogeneity assumption among studies was evaluated using a χ^2^-based *Q*-test. A *P*-value of > 0.10 for the *Q-*test indicated a lack of heterogeneity among studies. If significant heterogeneity was detected, the DerSimonian-Laird random-effects model was used; otherwise, the Mantel-Haenszel fixed-effects model was chosen [61, 62]. We investigated the relationship between *IFNG* *+874 T/A* (rs2430561) polymorphism and tuberculosis risk by testing the allelic effect (T vs. A), additive model (TT vs. AA and TA vs. AA), dominant model (T carriers vs. AA), recessive model (TT vs. A carriers), and overdominant model (TA vs. AA + TT). A sensitivity analysis was performed by successively omitting studies to assess the stability of the results. The departure of frequencies of the *IFNG* *+874 T/A* (rs2430561) polymorphism from the expected under HWE was assessed by Pearson's χ^2^ test, and *P* < 0.05 was considered significant [63]. Publication bias was assessed by both Egger's test and Begg's test, and a *P*-value < 0.05 was considered significant [64]. All statistical tests for this meta-analysis were performed using the Stata software version 11.0 (StataCorp LP, College Station, TX, USA). The power of our meta-analysis was calculated online using the website http://www.power-analysis.com/.

### Genotyping methods

Genotyping of the *IFNG* *+874 T/A* (rs2430561) SNP was conducted using methods described in the retrieved literature, such as polymerase chain reaction-restriction fragment length polymorphism (PCR-RFLP), polymerase chain reaction-amplification refractory mutation specific (PCR-ARMS), polymerase chain reaction-allele specific primers (PCR-ASP), polymerase chain reaction-sequence specific primer (PCR-SSP), polymerase chain reaction-allele-specific oligonucleotide (PCR-ASO), and amplification refractory mutation system-multi gene/multi primer polymerase chain reaction (ARMS-MG/MP-PCR).

## Abbreviations

TB: tuberculosis
PB: population-based
HB: hospital-based
MTB: Mycobacterium tuberculosis
IFNG: interferon-γ
Th1: T helper 1
PTB: pulmonary tuberculosis
EPTB: extra-pulmonary tuberculosis
HWE: Hardy-Weinberg equilibrium
MAF: Minor Allele Frequency
GWAS: Genome-wide association studies
NOS: Newcastle-Ottawa Scale
PCR-RFLP: polymerase chain reaction-restriction fragment length polymorphism
PCR-ARMS: polymerase chain reaction-amplification refractory mutation specific
PCR-ASP: polymerase chain reaction-allele specific primers
PCR-SSP: polymerase chain reaction-sequence specific primer
PCR-ASO: polymerase chain reaction-allele-specific oligonucleotide
ARMS-MG/MP-PCR: amplification refractory mutation system-multi gene/multi primer polymerase chain reaction
